# The Effect of *Citrus aurantium* on Non-Small-Cell Lung Cancer: A Research Based on Network and Experimental Pharmacology

**DOI:** 10.1155/2023/6407588

**Published:** 2023-01-23

**Authors:** Liangliang Yao, Xuan Zhang, Chaoming Huang, Yi Cai, Chunpeng (Craig) Wan

**Affiliations:** ^1^Affiliated Hospital of Jiangxi University of Chinese Medicine, Nanchang 330006, China; ^2^Guangzhou Municipal and Guangdong Provincial Key Laboratory of Molecular Target & Clinical Pharmacology, The NMPA and State Key Laboratory of Respiratory Disease, School of Pharmaceutical Sciences and the Fifth Affiliated Hospital, Guangzhou Medical University, Guangzhou 511436, China; ^3^College of Agronomy, Jiangxi Agricultural University, Jiangxi Key Laboratory for Post-Harvest Technology and Nondestructive Testing of Fruits & Vegetables, Nanchang 330045, China

## Abstract

**Purpose:**

To screen the main active components of *Citrus aurantium* through a network pharmacology approach, construct a component-disease target network, explore its molecular mechanism for the treatment of non-small-cell lung cancer (NSCLC), and validate it experimentally.

**Methods:**

The active ingredients in *Citrus aurantium* and the targets of *Citrus aurantium* and NSCLC were collected through the Traditional Chinese Medicine Systematic Pharmacology Database and Analysis Platform (TCMSP), GeneCards, and OMIM databases. The protein interaction network was constructed using the STRING database, and the component-disease relationship network graph was analyzed using Cytoscape 3.9.1. The Metascape database can be used for GO and KEGG enrichment analyses. The Kaplan-Meier plotter was applied for overall survival analysis of key targets of *Citrus aurantium* in the treatment of NSCLC. Real-time PCR (RT-PCR) and Western blotting were used to determine the mRNA and protein levels of key targets of *Citrus aurantium* for the treatment of NSCLC.

**Results:**

Five active ingredients of *Citrus aurantium* were screened, and 54 potential targets for the treatment of NSCLC were found, of which the key ingredient was nobiletin and the key targets are TP53, CXCL8, ESR1, PPAR-*α*, and MMP9. GO and KEGG enrichment analyses indicated that the mechanism of nobiletin in treating NSCLC may be related to the regulation of cancer signaling pathway, phosphatidylinositol-3 kinase (PI3K)/protein kinase B (Akt) signaling pathway, lipid and atherosclerosis signaling pathway, and neurodegenerative signaling pathway. The experimental results showed that nobiletin could inhibit the proliferation of NSCLC cells and upregulate the levels of P53 and PPAR-*α* and suppress the expression of MMP9 (*P* < 0.05).

**Conclusion:**

*Citrus aurantium* can participate in the treatment of NSCLC through multiple targets and pathways.

## 1. Introduction

Currently, lung cancer remains one of the most common cancers and has a very high mortality rate, accounting for approximately 18% of all cancer-related deaths [1]. According to the histological classification, lung cancer is divided into small-cell lung cancer (SCLC, 15% of all lung cancers) and non-small-cell lung cancer (NSCLS, 85% of all lung cancers) [2]. The process of NSCLC development is complex and diverse, involving multiple signaling pathways, such as PI3K/Akt signaling pathway, human matrix metalloproteinase 9 (MMP9), cell cycle protein-dependent kinase 1 (CDK1), and Wnt/*β*-catenin signaling pathway [3–6]. According to recent research results, the treatment of NSCLC is mainly based on Western medicine, including surgery, chemotherapy, radiotherapy, targeted therapy, and immunotherapy [7, 8]. However, these treatments are often accompanied by undesirable consequences such as susceptibility to recurrence and metastasis, poor prognosis, and high costs [9]. In addition, many targeted drugs used to treat NSCLC (e.g., EGFR-TKI and ALK-TKI), although they can prolong the survival of patients with advanced disease, the resistance of these drugs limits their long-term efficacy [10, 11]. Studies have shown that Chinese medicine can be very helpful in the adjuvant treatment or prognosis of NSCLC, reducing the adverse effects of EGFR-TKIs, improving disease-free survival (DFS), and having better tolerability [12, 13].

*Aurantii Fructus*, also known as ZhiQiao, is the dried unripe fruit of *Citrus aurantium* L. and its cultivated variants and is a traditional Chinese medicine. According to previous studies, *Citrus aurantium* has several potential pharmacological effects, such as promotion of intestinal motility [14], antidepressant effects [15, 16], antikidney stones [17], and antihepatotoxicity [18]. In addition, studies have shown that *Citrus aurantium* also has potential therapeutic effects on cardiovascular disease and cancer [19]. Weifuchun tablet is a proprietary Chinese medicine containing three Chinese herbs, namely, red ginseng, Isodon amethystoides, and *Citrus aurantium*, to relieve precancerous lesions of gastric cancer by regulating intestinal microbial balance and treating atrophy and intestinal metaplasia (IM) [20]. The above studies show that *Citrus aurantium* is a good anticancer herbal medicine, but the therapeutic effect and mechanism of action of *Citrus aurantium* on NSCLC have not been reported yet and deserve further study.

In this study, the protein interaction network between the active ingredients of *Citrus aurantium*, drug targets, and NSCLC-related disease genes was constructed through a network pharmacology approach to predict the potential targets and related pathways of *Citrus aurantium* for the treatment of NSCLC, which was then validated by cellular and molecular experiments, thus providing a theoretical basis for further clinical studies.

## 2. Materials and Methods

### 2.1. Collection of Active Ingredients and Targets of Citrus aurantium

The active ingredients of *Citrus aurantium* were obtained by searching “Zhiqiao” using the Traditional Chinese Medicine System Pharmacology Database and Analysis Platform (TCMSP) according to our previous study [21]. Then, we screened the list of ingredients with the criteria of oral bioavailability (OB) ≥ 30% and drug-likeness (DL) ≥ 0.18. Similarly, in the TCMSP database, the targets of active ingredients were screened in the list of relevant targets and a database of active ingredients and targets of *Citrus aurantium* was created. The target names were corrected using UniProt (https://www.uniprot.org/).

### 2.2. Collection of Disease Targets

Two online human-related gene databases, GeneCards (https://www.genecards.org/) and OMIM (https://omim.org/), were searched using the keyword “non-small-cell lung cancer” to obtain the NSCLC-related genes. The genes from the two databases were then integrated to create a database of NSCLC disease targets.

### 2.3. Venn Diagram of Genes Associated with Citrus aurantium and NSCLC

The target genes of *Citrus aurantium* and the target genes of NSCLC were uploaded on the Venny 2.1.0 online platform to map the Venn diagram and obtain the crossover genes of *Citrus aurantium* and NSCLC, i.e., the drug-disease cointeraction target genes.

### 2.4. Construction and Analysis of Protein Interaction Network

The information related to *Citrus aurantium* active ingredient and NSCLC target genes was imported into the network visualization software Cytoscape 3.9.1 (https://cytoscape3.9.1.org//) to construct a network diagram of the *Citrus aurantium* active ingredient-NSCLC target gene relationship. The data were analyzed using the CentiScaPe2.2 plug-in in Cytoscape 3.9.1 to calculate the nodes of each active ingredient in the network.

### 2.5. GO and KEGG Enrichment Analyses

The crossover genes of *Citrus aurantium* and NSCLC were entered into the STRING database (https://string-db.org/), and “Homo sapiens” was selected to map the protein interaction network. Subsequently, data analysis was performed using the CentiScaPe2.2 plug-in in Cytoscape 3.9.1 to screen for key targets in *Citrus aurantium* and NSCLC. Then, the crossover genes were entered into the Metascape database, “Homo sapiens” was selected, GO and KEGG enrichment analyses were performed, the data obtained from the analyses were stored, *P* values were calculated, and the relevant data were entered into the online platform Weishengxin (https://www.bioinformatics.com.cn/) to plot bubble plots.

### 2.6. Kaplan-Meier Analysis

The Kaplan-Meier plotter (https://kmplot.com/analysis/) was able to assess the impact of 54,000 genes on survival rates for 21 cancer types. Among them, the largest dataset includes breast, ovarian, lung, and gastric cancers [22]. In this study, it was used to assess the prognostic value of nobiletin-key target mRNA expression in NSCLC. Each of the 12 key targets was uploaded to the database to obtain a Kaplan-Meier survival plot, where the number of risks is shown below the main plot. When *P* < 0.05, it indicates that the results are significantly different. In this study, the threshold value with the best performance was used as the cutoff value, and “array quality control” was selected as “no filtered array quality.”

### 2.7. Drugs and Reagents

Dulbecco's modified Eagle medium (DMEM), fetal bovine serum (FBS), 0.25% trypsin-EDTA, and penicillin-streptomycin solution were purchased from Gibco (Logan, Utah, USA). Real-time PCR kits were purchased from Takara Co., Ltd. (Dalian, China). Rabbit anti-p53 and *β*-tubulin antibodies were purchased from CST Inc. (Boston, MA, USA). Chemiluminescent substrates were purchased from Pierce (Rockford, IL, USA).

### 2.8. Cell Culture

A549 cells were added to DMEM medium (Gibco) with 10% fetal bovine serum (FBS), 100 U/mL penicillin, and 100 U/mL streptomycin and maintained at 37°C, 95% air, and 5% CO_2_ in a humid environment. When the cells reached 80% to 90% fusion, the cells were digested with trypsin-EDTA (0.25%, Sigma), followed by passaging culture.

### 2.9. CCK-8 Analysis

Cell viability was assayed using Cell Counting Kit-8 (CCK-8). NSCLC cells in logarithmic growth phase were inoculated at 5 × 10^3^ per well into 96-well microplates and cultured for 24 h. Then, NSCLC cells were treated with different concentrations of nobiletin (0, 10, 20, and 40 *μ*M) for 12, 24, and 48 h. CCK-8 solution (10 mL) was added to each well and incubated for another 1 h. Finally, the absorbance of each well was measured at 450 nm by a multivolume spectrophotometer system (USA, BioTek Instruments Inc.).

### 2.10. Clone Formation Test

NSCLC cells (1 × 10^3^cells/well) in logarithmic growth phase were inoculated in 6-well culture plates and incubated at 37°C for 48 h. The cells were fixed with 4% paraformaldehyde (Solaibo, Beijing, China) for 10 min and stained with crystal violet (Sigma-Aldrich, China) for 30 min, and colonies containing more than 10 cells were observed under a microscope.

### 2.11. Real-Time PCR

Total RNA was extracted from cultured cells using TRIzol reagent (Invitrogen). mRNA was subsequently quantified, and cDNA was synthesized by reverse transcription, and mRNA levels of target genes were measured using a Bio-Rad quantitative PCR instrument. The specific primers used for RT-PCR are shown in Table 1, and GAPDH was used as an endogenous control. To ensure the validity and accuracy of the data, all reactions were performed three times.

### 2.12. Western Blotting

Total protein was extracted from the cells using RIPA lysis buffer, and the extracted protein was then quantified using the BCA protein quantification kit according to the manufacturer's instructions. Subsequently, equivalent proteins were separated on 10% SDS-PAGE, then transferred to poly(vinylidene fluoride) (PVDF) membranes and blocked against nonspecific antibodies; primary antibodies were added, incubated for 1-2 h at room temperature, and then incubated with secondary antibodies for 1 h at room temperature; and target protein expression was detected by chemiluminescence, and the bands were analyzed in grayscale by the ImageJ software.

### 2.13. Statistical Analysis

All data were expressed as mean ± standard deviation, and statistical analysis was performed using the SPSS 13.0 statistical software. One-way analysis of variance (ANOVA) was used for comparison of means between multiple groups. Differences were considered statistically significant at *P* < 0.05.

## 3. Results

### 3.1. Establishment of a Database of Active Ingredients and Targets of *Citrus aurantium*

Through the TCMSP database, 17 active ingredients of *Citrus aurantium* were available, and the list of ingredients was screened by oral bioavailability (OB) ≥ 30% and adult drug similarity (DL) ≥ 0.18, and finally, five highly active ingredients were obtained (Table 1).

**Figure 1:**
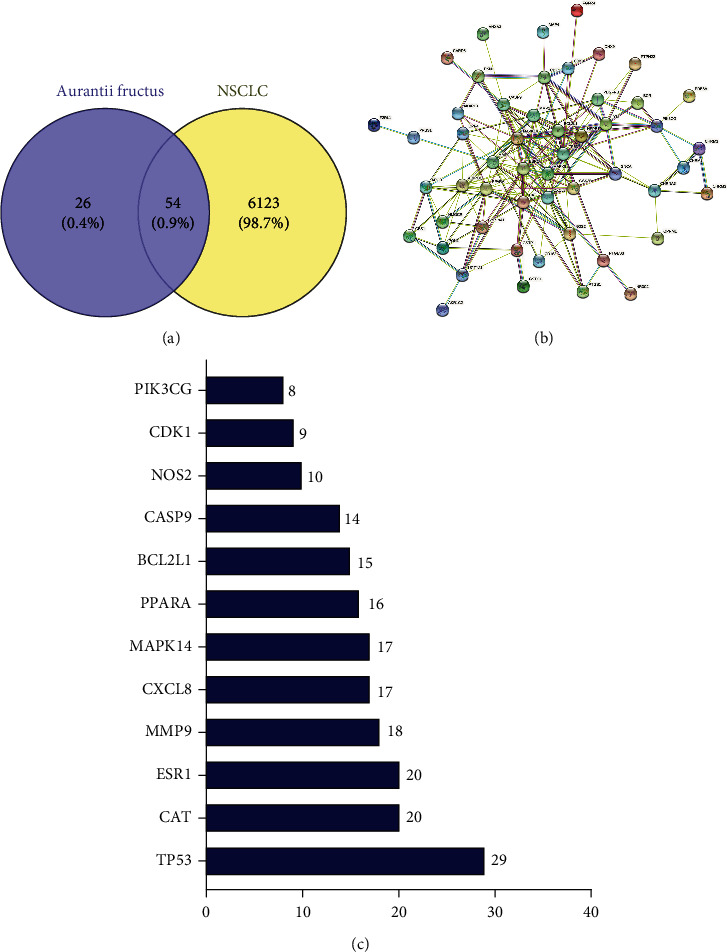
Network diagram of potential target genes and PPI of *Citrus aurantium* for the treatment of NSCLC. (a) Venny results of potential target genes of *Citrus aurantium* for the treatment of NSCLC. (b) PPI network diagram of 51 target genes. (c) Key targets of *Citrus aurantium*-NSCLC.

Similarly, 124 relevant targets for five highly active ingredients were available in the TCMSP database. Target names were corrected and deduplicated using UniProt to finally obtain 80 relevant target genes for *Citrus aurantium*. Based on GeneCards and OMIM databases, a total of 6177 target genes related to NSCLC were obtained. The Venn diagram of *Citrus aurantium* and NSCLC was drawn using the Venny 2.1.0 online platform, and 54 crossover genes were obtained by analysis (Table 2 and Figure 1(a)).

### 3.2. Protein Interaction Network Analysis

The protein interaction network graph of *Citrus aurantium* with NSCLC can be obtained by entering the crossover genes into the STRING online platform and hiding the free nodes outside the network (Figure 1(b)). After analysis, there are 52 nodes and 195 edges with an average node number of 7.22. In the network diagram, network nodes represent proteins, edges represent protein-protein associations, and different color edges indicate different meanings: light blue indicates from selected databases, purple indicates experimentally determined, and these two are known; green indicates gene neighborhood, red indicates gene fusion, and dark blue indicates gene cooccurrence, and these three are predicted; there are also yellow that indicates text mining, black indicates coexpression, and white indicates protein homology. The protein interaction network map was imported into Cytoscape 3.9.1, and the data were analyzed using the CentiScaPe2.2 plug-in, and 12 key targets (Figure 1(c)) were obtained after filtering based on the mediator centrality (BC), closeness centrality (CC), and degree centrality (DC) parameters. As can be seen from the figure, the top seven genes with node degree values are TP53, CAT, ESR1, MMP9, CXCL8, MAPK14, and PPAR-*α*, indicating that these genes are in key positions in the protein interaction network.

### 3.3. Construction and Analysis of Drug Component-Disease Target Networks

**Figure 2:**
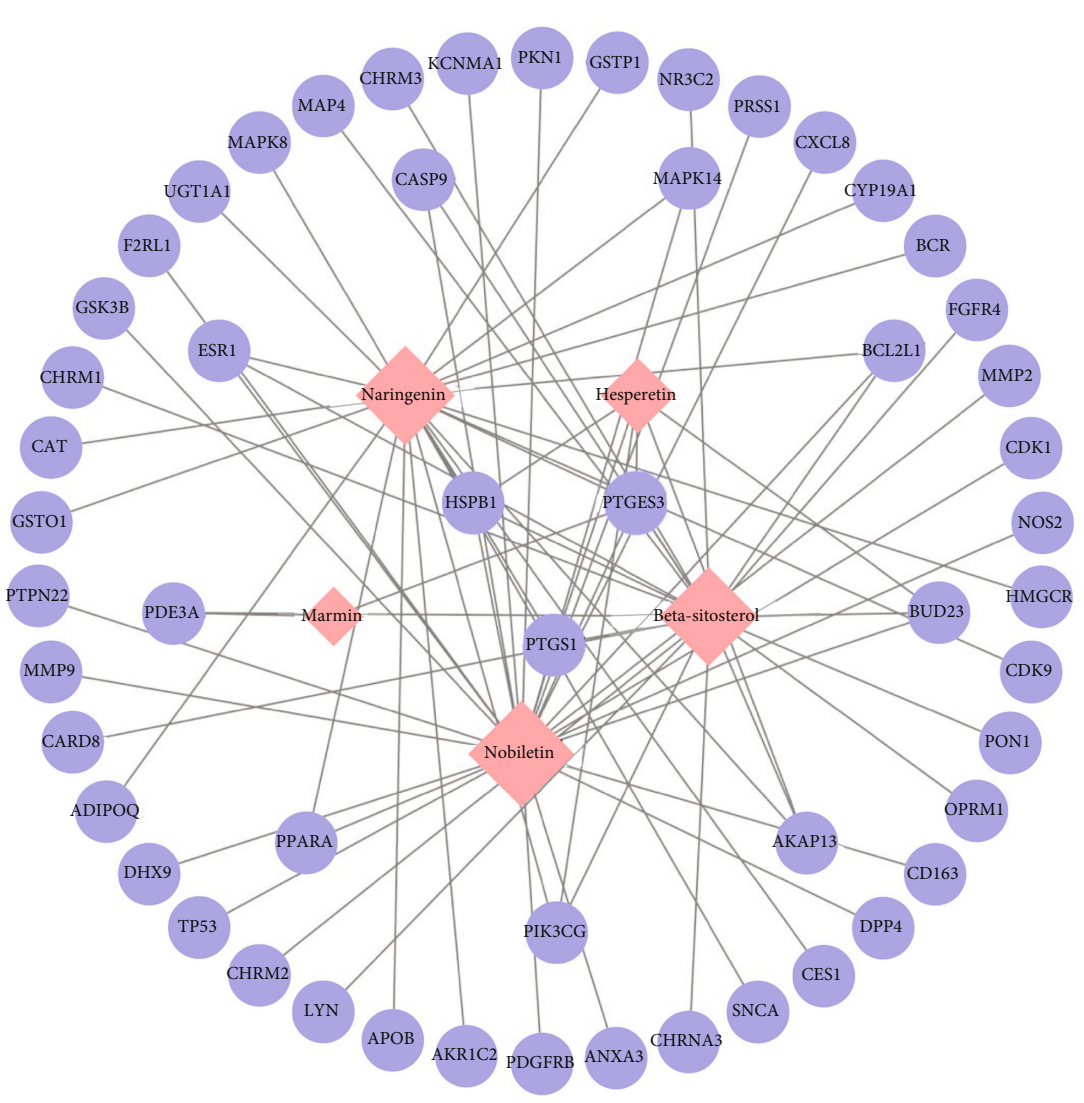
*Citrus aurantium*-NSCLC potential target gene network.

The information related to the active ingredients of *Citrus aurantium* and the crossover genes between *Citrus aurantium* and NSCLC was imported into Cytoscape 3.9.1 to construct a *Citrus aurantium* component-NSCLC target network map (Figure 2). Using one of the analyses, CentiScaPe2.2, the key component of *Citrus aurantium* for the treatment of NSCLC was finally obtained as nobiletin based on the mesocentricity (BC), closeness centrality (CC), and degree centrality (DC) parameters. The results showed that nobiletin was higher than the other four components, suggesting that nobiletin in *Citrus aurantium* is a key component in the treatment of NSCLC (Table 3).

### 3.4. GO and KEGG Enrichment Analyses

**Figure 3:**
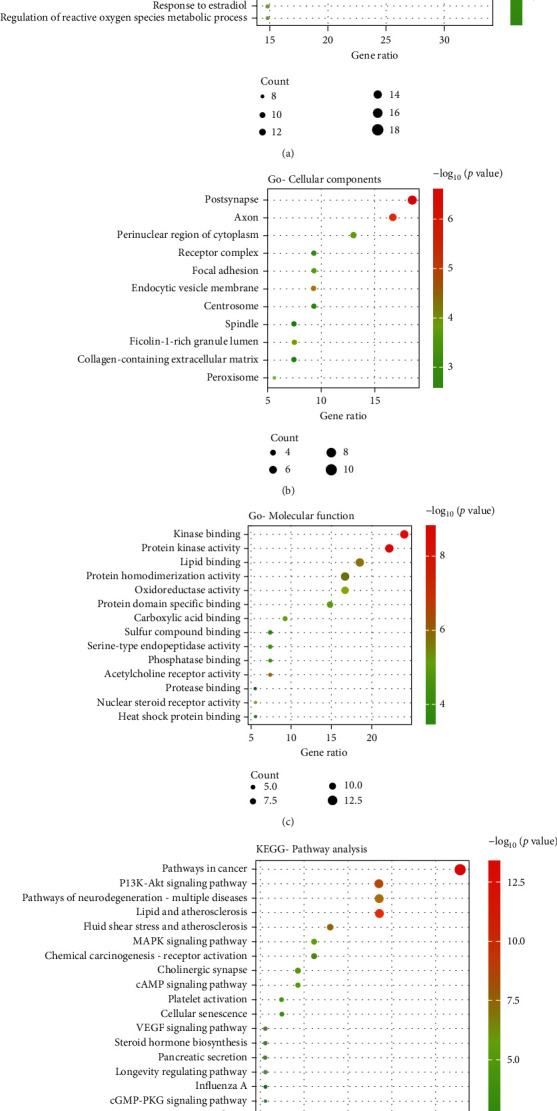
GO and KEGG analyses of potential target genes of *Citrus aurantium* in NSCLC. GO analysis of biological processes (a), molecular functions (b), and cellular components (c) of potential target genes of *Citrus aurantium* in NSCLC. (d) KEGG enrichment analysis of potential target gene signaling pathway of *Citrus aurantium* in NSCLC.

The *Citrus aurantium*-NSCLC crossover genes were entered into the Metascape database for GO and KEGG enrichment analyses. Based on *P* < 0.01, a minimum number of 3, and enrichment factor > 1.5, GO analysis yielded 20 biological processes, 14 molecular functions, and 11 cellular compositions that are component targets of *Citrus aurantium* for the treatment of NSCLC (Figures 3(a)–3(c)). The results showed that cellular responses to organic cyclic compounds (target number 18), cellular responses to lipids (target number 18), negative regulation of intracellular signaling (target number 15), responses to lipopolysaccharides (target number 14), and responses to exogenous stimuli (target number 14) were significantly enriched in *Citrus aurantium* for the treatment of NSCLC, indicating that *Citrus aurantium* is able to treat NSCLC through multiple biological pathways. 140 pathways were obtained by KEGG enrichment analysis (*P* < 0.01), and 18 pathways with the highest correlation to NSCLC were obtained by screening (Figure 3(d)). The results showed the highest enrichment in tumor-related signaling pathway (target number 15), lipid and atherosclerosis signaling pathway (target number 10), PI3K-Akt signaling pathway (target number 10), and neurodegenerative signaling pathway (target number 10), indicating that *Citrus aurantium* can treat NSCLC through these pathways.

### 3.5. Prognostic Value of Key Targets

**Figure 4:**
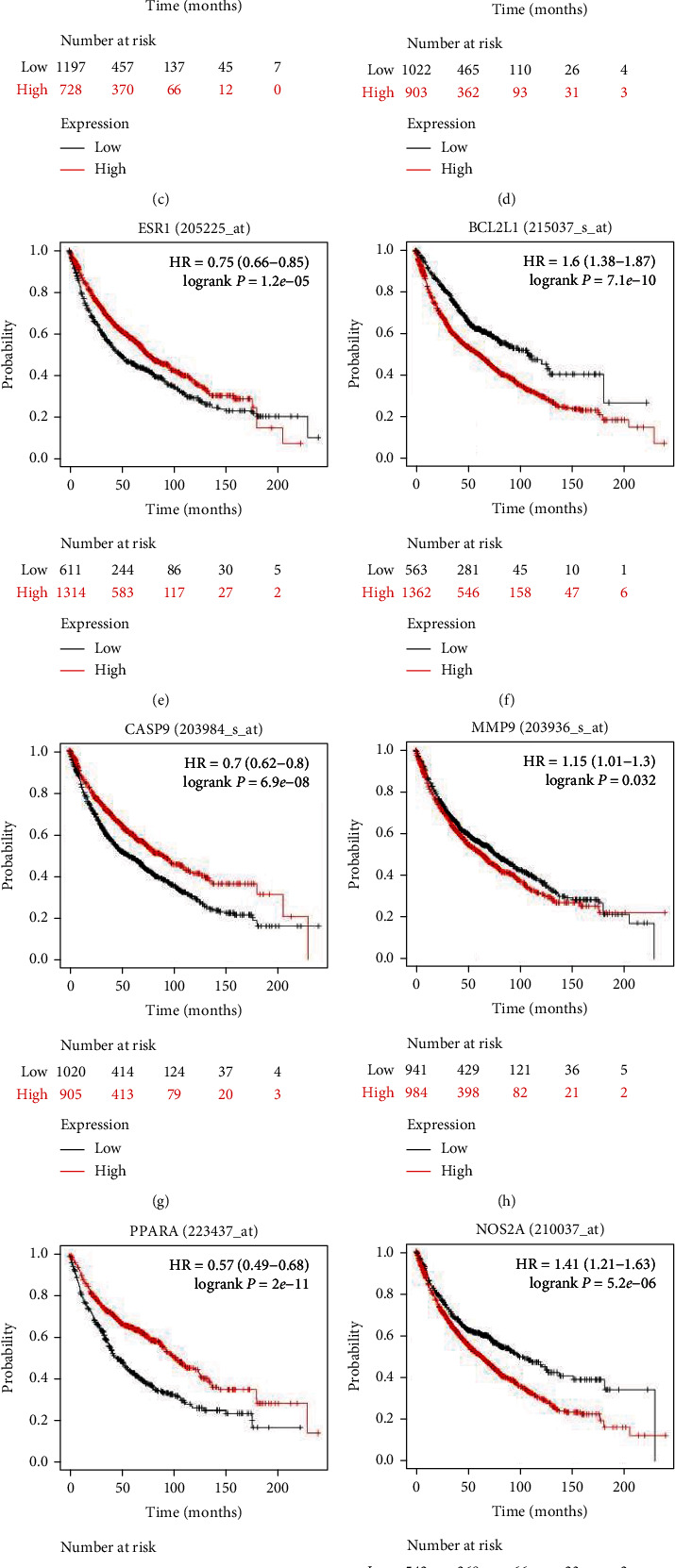
Prognostic characteristics of 12 key targets in nobiletin-NSLC. Overall survival (OS) survival curves for key targets were plotted using the Kaplan-Meier plotter database (*P* < 0.05, *n* = 2437). (a) TP53; (b) CAT; (c) MAPK14; (d) MDCNF(CXCL8); (e) ESR1; (f) BCL2L1; (g) CASP9; (h) MMP9; (i) PPARA; (j) NOS2A(NOS2); (k) PIK3CG; black line indicates low expression, and red line indicates high expression.

Survival analysis was performed by the Kaplan-Meier plotter to evaluate the expression and prognosis of 12 key targets in NSCLC. As shown in Figure 4, TP53 (HR = 1.44 [1.27-1.64], logrank *P* = 1.4*e* − 08), CXCL8 (HR = 1.3 [1.15-1.47], logrank *P* = 4.6*e* − 05), BCL2L1 (HR = 1.38 [1.38-1.87], logrank *P* = 7.1*e* − 10), MMP9 (HR = 1.15 [1.01-1.3], logrank *P* = 0.032), and NOS2 (HR = 1.41 [1.21-1.63], logrank *P* = 5.2*e* − 06) high mRNA expressions were associated with significantly shorter OS in all NSCLC patients (*P* < 0.05); i.e., survival was lower in the high expression group than in the low expression group. In contrast, CAT (HR = 0.48 [0.41-0.56], logrank *P* < 1*e* − 16), ESR1 (HR = 0.75 [0.66-0.85], logrank *P* = 1.2*e* − 05), MAPK14 (HR = 0.63 [0.55-0.72], logrank *P* = 2.9*e* − 11), CASP9 (HR = 0.7 [0.62-0.8], logrank *P* = 6.9*e* − 08), PIK3CG (HR = 0.39 [0.31-0.48], logrank *P* < 1*e* − 16), and PPAR-*α* (HR = 0.57 [0.49-0.68], logrank *P* = 2*e* − 11) mRNA high expressions were associated with significantly improved OS in all NSCLC patients (*P* < 0.05); i.e., survival was higher in the high expression group than in the low expression group. In addition, CDK1 lacked relevant data.

### 3.6. Effect of Nobiletin on the Proliferation of NSCLC A549 Cells

**Figure 5:**
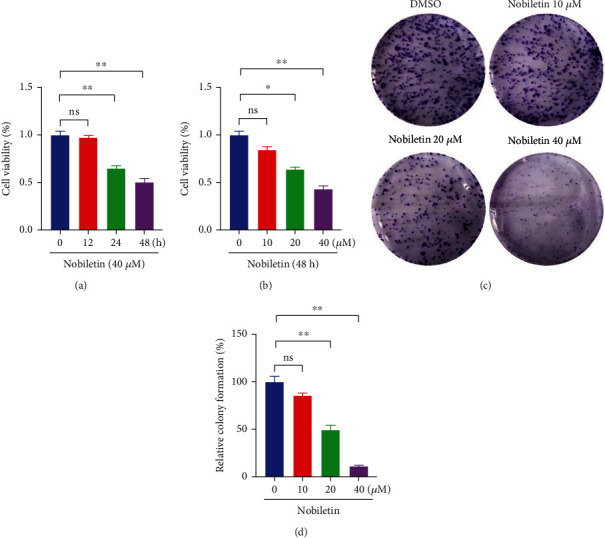
Effect of nobiletin on the proliferation of NSCLC cells. (a) NSCLC cells were treated with 40 *μ*M nobiletin, and the effect of nobiletin on the proliferation of NSCLC cells at 0, 12, 24, and 48 h was examined by CCK-8 analysis. (b) NSCLC cells were treated with different concentrations of nobiletin for 48 h, and the effect of nobiletin on the proliferation of NSCLC cells was detected by CCK-8 analysis. (c, d) Cell proliferation was assessed by colony formation assay after treatment of NSCLC cells with different concentrations of nobiletin. ^∗^*P* < 0.05 compared with the untreated group, *n* = 5.

To assess whether nobiletin could inhibit the growth of NSCLC cells, we treated NSCLC cells with different concentrations of nobiletin (0, 10, 20, and 40 *μ*M) for 12, 24, and 48 h and then measured cell viability using the CCK8 assay. The results showed that nobiletin inhibited the proliferation of NSCLC cells in a dose-dependent manner (Figures 5(a) and 5(b)). In addition, clonogenesis assays confirmed that nobiletin inhibited the colony-forming ability of NSCLC cells in a dose-dependent manner compared to untreated cells (Figures 5(c) and 5(d)). The above results suggest that nobiletin inhibits the proliferation of NSCLC A549 cells.

### 3.7. Effect of Nobiletin on Key Targets in NSCLC

**Figure 6:**
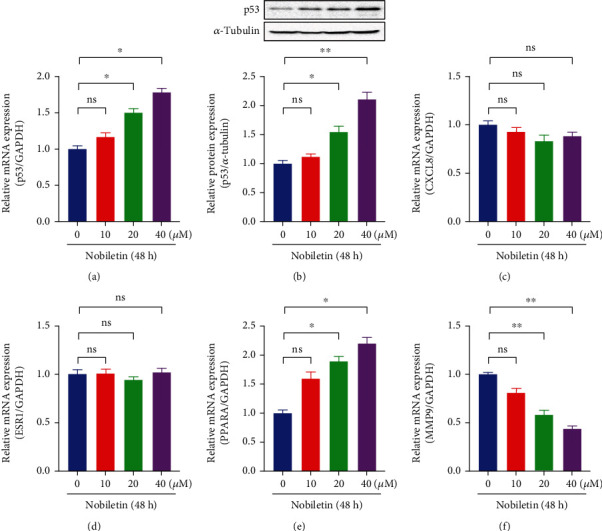
Effect of nobiletin on the expression of key target genes. NSCLC cells were treated with 0, 10, 20, and 40 *μ*M nobiletin for 48 h. Real-time PCR was performed to detect the mRNA expression of (a) TP53, (c) CXCL8, (d) ESR1, (e) PPARA, and (f) MMP9. (b) Protein expression of TP53 was detected by protein immunoblotting. ^∗^*P* < 0.05 compared with the untreated group, *n* = 5.

To demonstrate the effect of nobiletin on key targets in NSCLC, we treated A549 cells with different concentrations of nobiletin (0, 10, 20, and 40 *μ*M) for 48 h and detected the mRNA expression of p53, ESR1, MMP9, CXCL8, and PARPA by real-time PCR (Table 4). The protein expression level of p53 was detected by Western blotting method. The results showed that nobiletin dose-dependently increased the mRNA and protein expression of P53 (Figures 6(a) and 6(b)). In addition, we also found that nobiletin was able to increase the mRNA expression of PPAR-*α* and inhibit the mRNA level of MMP9 (Figures 6(e) and 6(f)). Moreover, nobiletin had no significant effect on the expression of CXCL8 and ESR1 (Figures 6(c) and 6(d)).

## 4. Discussion

Lung cancer is one of the malignant tumors with the highest incidence and mortality rate worldwide, but the current clinical treatment is not satisfactory [3]. In this study, we explored the potential therapeutic targets and major molecular mechanisms of *Citrus aurantium* for the treatment of NSCLC based on network pharmacology analysis and bioinformatics. Cyberpharmacology combines system biology and pharmacology to analyze and explore the multichannel regulation of signaling pathways through high-throughput sequencing, genomics, and other technologies to find therapeutic targets and signaling pathways for diseases [23]. Based on network pharmacology, we collected 5 active ingredients, 80 targets, and 177 disease targets of *Citrus aurantium* and obtained 54 crossover genes in this study. The results of protein interaction network combined with enrichment analysis were used to obtain nobiletin, a key compound of *Citrus aurantium* for the treatment of NSCLC, and five key targets: TP53, CXCL8, ESR1, PPAR-*α*, and MMP9. GO bioprocess analysis showed that *Citrus aurantium* can treat NSCLC as long as the biological processes such as cellular response through organic cyclic compounds, cellular response to lipids, and negative regulation of intracellular signal transduction. KEGG signaling pathway enrichment analysis showed that the main pathways of *Citrus aurantium* for the treatment of NSCLC were tumor-associated signaling pathway, lipid and atherosclerosis signaling pathway, PI3K-Akt signaling pathway, neurodegenerative signaling pathway, and MAPK signaling pathway.

The development and progression of NSCLC is synergistically regulated by multiple extracellular and intracellular signals. The TP53 gene is located on human chromosome 17 and is involved in the encoding of the p53 protein, which inhibits cancer formation by interacting with various related signaling pathways [24]. Activated p53 protein transcriptionally regulates hundreds of genes involved in multiple biological processes, including DNA repair, cell cycle arrest, cell growth, cell division, apoptosis, senescence, autophagy, and metabolism, thereby mediating cancer suppression [25–28]. NF-*κ*B and Bax proteins are upstream and downstream targets of p53, respectively, and play important roles in cell growth. Guo et al. [29] showed that PAQR3 inhibits the development and progression of NSCLC through the NF-*κ*B/p53/Bax signaling pathway. In addition, it was found that p53 expression levels positively correlated with apoptosis in NSCLC tissues and inhibited the proliferation of lung cancer cells by increasing apoptosis, thereby inhibiting tumor growth and delaying the development of NSCLC [30]. MMP9, a member of the matrix metalloproteinase family (MMPs), is located on human chromosome 20 and is one of the most important enzymes in the breakdown of the extracellular matrix, playing a key role in the invasion and metastasis of cancer [31]. Previous studies have shown that MMP9 expression can be downregulated by reducing AKT/mTOR levels that promote H3K27Ac and H3K56A on the MMP9 promoter region, thereby inhibiting the proliferation and metastasis of triple-negative breast cancer [32], and that MMP-9 can also promote the invasion and migration of gastric cancer cells through the ERK pathway [33]. In addition, a study by Zhang et al. [34] found that the expression level of MMP9 was significantly higher in lung cancer patients than in normal subjects, and the higher the expression of MMP9, the worse the survival status of lung cancer patients. In the present work, we found that nobiletin significantly increased the expression level of P53 and inhibited the expression of MMP9 in A549 cells, suggesting that P53 and MMP9 may be downstream targets of nobiletin in regulating NSCLC.

CXCL8 is a proinflammatory CXC chemokine, called interleukin-8 (IL-8), involved in tumor angiogenesis to promote tumorigenesis and metastasis [35]. The Kaplan-Meier survival analysis showed that high IL-8 expression was associated with a poorer prognosis in NSCLC patients (*P* < 0.05, Figure 4(b)). CXCL8 interacts with CXCL1 and CXCL2 to promote the secretion of multiple proinflammatory, angiogenic, and immunomodulatory factors (including MMP and VEGF) by neutrophils, thereby promoting tumor metastasis in patients with NSCLC [36, 37]. Yan et al. [38] showed that IL-8 is highly expressed in lung cancer and suggested that it could be a potential biomarker for lung cancer and has strong diagnostic properties for lung cancer. In another study, the methyltransferase SETD2 was found to inhibit tumor growth and metastasis through STAT1-IL-8 signaling-mediated epithelial-mesenchymal transition in lung adenocarcinoma [39]. ESR1 is localized on chromosome 6 and belongs to the transcriptional activator superfamily, whose protein product is a transcription factor primarily involved in encoding the estrogen receptor. It was shown that hypermethylation of ESR1 was detected only in lung tumors but not in adjacent normal lung tissue, suggesting that ESR1 hypermethylation may be associated with the development of lung cancer. Assessment of p16 and ESR1 methylation in blood facilitates early diagnosis of lung cancer, and these methylation genes may be biomarkers for early lung cancer [40, 41]. It has been shown that ESR1 signaling plays a biological role in both epithelial and mesenchymal cells in the lung and that ESR1 may promote lung cancer by acting directly on precancerous or tumor cells or indirectly on lung fibroblasts [42]. However, in the present study, we did not find that nobiletin was able to affect the levels of ESR1 and CXCL8.

Peroxisome proliferator-activated receptor alpha (PPAR-*α*) is a ligand-activated nuclear receptor that regulates transcription of target genes associated with lipid homeostasis, differentiation, and inflammation in a variety of ways [43–45]. Luo et al. [46] showed that intestinal PPAR-*α* deficiency in mice increased azomethane- (AOM-) induced colon tumorigenesis and tumor growth. In addition, they demonstrated by IHC staining that the PPAR-*α*-DNMT1/PRMT6-p21/p27 regulatory pathway may also play a role in the early stages of human colorectal carcinogenesis. Previous studies found that apatinib exerts its antitumor effects through the induction of ketogenesis and demonstrated that this tumor-suppressive effect is PPAR-*α*-dependent [47]. In another study, fenofibrate, a PPAR-*α* agonist, was found to alleviate resistance to gefitinib in NSCLC cell lines by modulating the PPAR-*α*/AMPK/AKT/FoxO1 signaling pathway and demonstrated that the increased antiproliferative effect of fenofibrate was abolished when PPAR-*α* was silenced [48]. In addition, several studies have shown that activation of PPAR-*α* can inhibit lung cancer growth and metastasis by downregulating cytochrome P450 arachidonic acid cyclooxygenase (Cyp2c) [49, 50]. In the present study, we found that nobiletin significantly increased the expression of PPAR-*α*, suggesting that PPAR-*α* may also be involved in the regulatory role of nobiletin in NSCLC.

## 5. Conclusion

In conclusion, we found through our study that nobiletin in *Citrus aurantium* may be a key active ingredient in the treatment of NSCLC. In addition, further analysis showed that nobiletin inhibited the development of NSCLC through targets such as TP53, CXCL8, ESR1, PPAR-*α*, and MMP9 and related signaling pathways. Through pharmacological experiments, we verified that nobiletin inhibited the proliferation of NSCLC A549 cell line and affected the expression of P53, PPAR-*α*, and MMP9. In future experiments, we will further clarify the downstream targets of nobiletin that regulate the proliferation of NSCLC through gene overexpression and silencing. This study provides a reference for further research on the mechanism of action of *Citrus aurantium* in the treatment of NSCLC.

## Figures and Tables

**Table 1:** Characteristics of active ingredients in *Citrus aurantium*.

No.	Mol ID	Molecule name	Molecular weight	OB (%)	DL
1	MOL013381	Marmin	332.43	38.23	0.31
2	MOL002341	Hesperetin	302.3	70.31	0.27
3	MOL000358	Beta-sitosterol	414.79	36.91	0.75
4	MOL004328	Naringenin	272.27	59.29	0.21
5	MOL005828	Nobiletin	402.43	61.67	0.52

OB: oral bioavailability; DL: drug-likeness.

**Table 2:** 54 potential target genes of *Citrus aurantium* therapy for NSCLC.

No.	Target	Symbol	UniProt ID
1	Prostaglandin G/H synthase 1	PTGS1	P23219
2	Prostaglandin G/H synthase 2	PTGES3	Q15185
3	Heat shock protein HSP 90	HSPB1	P04792
4	Phosphatidylinositol-4,5-bisphosphate 3-kinase catalytic subunit, gamma isoform	PIK3CG	P48736
5	mRNA of PKA catalytic subunit C-alpha	AKAP13	Q12802
6	Nuclear receptor coactivator 1/nuclear receptor coactivator 2	BUD23	O43709
7	Progesterone receptor	NR3C2	P08235
8	Muscarinic acetylcholine receptor M3	CHRM3	P20309
9	Muscarinic acetylcholine receptor M1	CHRM1	P11229
10	CGMP-inhibited 3′,5′-cyclic phosphodiesterase A	PDE3A	Q14432
11	Muscarinic acetylcholine receptor M2	CHRM2	P08172
12	Neuronal acetylcholine receptor subunit alpha-2	CHRNA3	P32297
13	Mu-type opioid receptor	OPRM1	P35372
14	Cytochrome P450-cam	FGFR4	P22455
15	Apoptosis regulator Bcl-2/apoptosis regulator BAX	BCL2L1	Q07817
16	Caspase-9	CASP9	P55211
17	Transcription factor AP-1	ESR1	P03372
18	Caspase-8	CARD8	Q9Y2G2
19	Transforming growth factor beta-1	LYN	P07948
20	Serum paraoxonase/arylesterase 1	PON1	P27169
21	Microtubule-associated protein 2	MAP4	P27816
22	Transcription factor p65	CDK9	P50750
23	RAC-alpha serine/threonine-protein kinase	BCR	P11274
24	Mitogen-activated protein kinase 1/mitogen-activated protein kinase 3	MAPK14	Q16539
25	Bcl2 antagonist of cell death	MAPK8	P45983
26	Catalase	CAT	P04040
27	Peroxisome proliferator-activated receptor gamma	PPARA	Q07869
28	Apolipoprotein B-100	APOB	P04114
29	Phospholipase B1, membrane-associated	SNCA	P37840
30	3-Hydroxy-3-methylglutaryl-coenzyme A reductase	HMGCR	P04035
31	Cytochrome P450 19A1	CYP19A1	P11511
32	Glutathione S-transferase P	GSTP1	P09211
33	UDP-glucuronosyltransferase 1-1	UGT1A1	P22309
34	Glutathione reductase, mitochondrial	GSTO1	P78417
35	Adiponectin	ADIPOQ	Q15848
36	Aldo-keto reductase family 1 member C1	AKR1C2	P52895
37	Liver carboxylesterase 1	CES1	P23141
38	Nitric oxide synthase, inducible	NOS2	P35228
39	Thrombin	CXCL8	P10145
40	Androgen receptor	PKN1	Q16512
41	Coagulation factor Xa/coagulation factor VII	F2RL1	P55085
42	mRNA of protein-tyrosine phosphatase, nonreceptor type 1	PTPN22	Q9Y2R2
43	DNA topoisomerase II	DHX9	Q08211
44	Estrogen receptor beta	PDGFRB	P09619
45	Dipeptidyl peptidase IV	DPP4	P27487
46	Serine/threonine-protein kinase Chk1	CDK1	P06493
47	Trypsin-1	PRSS1	P07477
48	Calcium-activated potassium channel subunit alpha 1	KCNMA1	Q12791
49	Glycogen synthase kinase-3 beta	GSK3B	P49841
50	Matrix metalloproteinase-9	MMP9	P14780
51	Cellular tumor antigen p53	TP53	P04637
52	Cytosolic phospholipase A2	ANXA3	P12429
53	Scavenger receptor cysteine-rich type 1 protein M130	CD163	Q86VB7
54	Ephrin type-B receptor 2	MMP2	P08253

**Table 3:** The list of key active components in *Citrus aurantium* dependent on the centrality of a node.

No.	Molecule name	Degree	Closeness unDir	Betweenness unDir
1	Hesperetin	6	0.006329114	36.36140351
2	Beta-sitosterol	21	0.0078125	1319.583584
3	Naringenin	23	0.008064516	1510.188889
4	Nobiletin	26	0.008474576	1761.830409
5	Marmin	2	0.006024096	10.03571429

**Table 4:** Primer sequences for qRT-PCR.

Primer	Sequences
P53	Forward:5′-GTTTCCGTCTGGGCTTCT-3′
	Reverse: 5′-CAACCTCCGTCATGTGCT-3′
CXCL8	Forward: 5′-ACATACTCCAAACCTTTCC-3′
	Reverse: 5′-AACTTCTCCACAACCCTC-3′
ESR1	Forward: 5′-AGGGAAGTATGGCTATGGAA-3′
	Reverse: 5′-ACTGGTTGGTGGCTGGAC-3′
PPARA	Forward: 5′-CATCGGCGAGGATAGTTC-3′
	Reverse: 5′-TGAAAGCGTGTCCGTGAT-3′
MMP9	Forward: 5′-TCCCTGGAGACCTGAGAACC-3′
	Reverse: 5′-GCCACCCGAGTGTAACCAT-3′
GAPDH	Forward: 5′-AGGAGTAAGAAACCCTGGAC-3′
	Reverse: 5′-CTGGGATGGAATTGTGAG-3′

## Data Availability

The data used to support the findings of this study are available from the corresponding authors upon reasonable request.
